# Microbiology of diabetic foot infections: from Louis Pasteur to ‘crime scene investigation’

**DOI:** 10.1186/s12916-014-0232-0

**Published:** 2015-01-07

**Authors:** Anne Spichler, Bonnie L Hurwitz, David G Armstrong, Benjamin A Lipsky

**Affiliations:** Current address; Department of Medicine, University of Arizona Health Science Center, 1501 N. Campbell Ave., Tucson, AZ 85724 USA; Department of Agricultural and Biosystems Engineering, University of Arizona, Tucson, Arizona USA; Department of Surgery, Southern Arizona Limb Salvage Alliance (SALSA), University of Arizona Health Sciences Center, Tucson, Arizona USA; Service of Infectious Diseases, Geneva University Hospitals and Department of Medicine, University of Geneva, Geneva, Switzerland; Division of Medical Sciences, Green Templeton College, University of Oxford, 79 Stone Meadow, Oxford, OX2 6TD UK

**Keywords:** Molecular diagnostics, Diabetic foot infection, Microbiology, Metagenomics, High-throughput sequencing

## Abstract

Were he alive today, would Louis Pasteur still champion culture methods he pioneered over 150 years ago for identifying bacterial pathogens? Or, might he suggest that new molecular techniques may prove a better way forward for quickly detecting the true microbial diversity of wounds? As modern clinicians faced with treating complex patients with diabetic foot infections (DFI), should we still request venerated and familiar culture and sensitivity methods, or is it time to ask for newer molecular tests, such as 16S rRNA gene sequencing? Or, are molecular techniques as yet too experimental, non-specific and expensive for current clinical use? While molecular techniques help us to identify more microorganisms from a DFI, can they tell us ‘who done it?’, that is, which are the causative pathogens and which are merely colonizers? Furthermore, can molecular techniques provide clinically relevant, rapid information on the virulence of wound isolates and their antibiotic sensitivities? We herein review current knowledge on the microbiology of DFI, from standard culture methods to the current era of rapid and comprehensive ‘crime scene investigation’ (CSI) techniques.

## Introduction

***“[H]ave confidence in those powerful and safe methods, of which we do not yet know all the secrets.” “I am on the verge of mysteries, and the veil is getting thinner and thinner.”***

***-Louis Pasteur* [**1**]**

This review is designed to address how to define diabetic foot infections (DFI) and to discuss the current understanding of the best way to evaluate the microbiology of these complex infections. We also summarize new data on the DFI microbiota, including the methodological metamorphosis of our understanding from Pasteur’s time to the current (and near-future) era. Further, we present information on newly available molecular microbiology methods and examine innovative technologies that may be used in the near future for defining pathogens in DFI.

## Review

### Wound infection: definition, process and prognosis

Foot wounds are an increasingly common problem in people with diabetes and now constitute the most frequent diabetes-related cause of hospitalization [2]. People with diabetes have about a 25% chance of developing a foot ulcer in their lifetime [3], about half of which are clinically infected at presentation [2,4]. DFIs cause substantial morbidity and at least one in five results in a lower extremity amputation [5]. Amputation is even more likely when DFI and foot ischemia coexist. [4,6] In fact, DFIs are now the predominant proximate trigger for lower extremity amputations worldwide [7].

The pathophysiology of foot infections in persons with diabetes is quite complex, but their prevalence and severity are largely a consequence of host-related disturbances (immunopathy, neuropathy and arteriopathy) and secondarily, pathogen-related factors (virulence, antibiotic-resistance and microbial load) [2,8]. Typically, an insensate, deformed foot develops an ulcer when some form of trauma disrupts the protective skin envelope. The underlying subcutaneous tissues then quickly become colonized with bacteria, which may lead to infection, often initially clinically unapparent [7]. Infection is defined by overgrowth of microorganisms within a wound that promotes deleterious inflammation or tissue destruction [9]. Infection usually begins as a local process, manifested by the classic signs and symptoms of inflammation (redness, warmth, pain, tenderness, induration) [10]. If not controlled, infection typically spreads—mostly often contiguously—to deeper tissues. A host systemic inflammatory response syndrome (for example, fever, chills, hypotension, tachycardia, delirium, leukocytosis) may accompany this process [10]. In some patients, especially those with peripheral neuropathy or vasculopathy, these symptoms and signs may be diminished [11,12], leading some to advocate defining infection by the presence of ‘secondary’ findings, such as foul odor, friable or discolored granulation tissue and rim undermining [13].

Some wound specialists believe that the presence of a high concentration of microorganisms (usually defined as >10^5^ colony forming units [CFU] per gram of host tissue [14]) in the absence of clinical evidence of infection represents ‘increased bioburden’ or ‘critical colonization.’ They assert this may indicate wound infection [15,16], or at least a degree of colonization that impairs wound healing [12], and may overwhelm host defenses without triggering a generalized immunological reaction [8]. There is, however, no agreed means to define critical colonization, no routine laboratory availability for quantitative bacteriology and no convincing evidence of its association with adverse clinical outcomes, for example, failure of healing or development of overt infection.

One study did find that in neuropathic diabetic foot ulcers (DFUs) there was a significant inverse relationship between exudate CFU count and rate of wound healing [17]. But, in a cross-sectional study of 64 patients with a non-ischemic DFU, no single sign or symptom generally recognized as suggestive of infection, or any combination of them, correlated well with the quantitative microbial load [16]. Unfortunately, this lack of correlation does not clarify whether clinical or microbiological results are most useful in defining infection. It may be that the presence of specific types, or combinations, of bacteria, or their acquisition of certain virulence factors, leads to clinical infection [12]. A prospective study of 77 patients with a neuropathic DFU and no clinical signs of infection found that none of the three dimensions of bioburden (that is, microbial load, microbial diversity and presence of potential pathogens) correlated with DFU outcomes. Some limitations of this study included the fact that specimens were obtained from the ulcer by swab (using ‘Levine’s technique’) and bioburden analysis was done by culture-based methods [18].

### Diabetic foot infection: bacteriology

Because many different organisms, alone or in combination, can cause a DFI, selecting the most appropriate antibiotic therapy requires defining the specific causative pathogens [8,10,12]. Clinicians should avoid antibiotic therapy that is unnecessary, overly broad-spectrum or excessively prolonged, as it may cause drug-related adverse effects, incurs financial cost and encourages antimicrobial resistance [10].

Aerobic, Gram-positive cocci are the predominant organisms responsible for acute DFI, with *Staphylococcus aureus* the most commonly isolated pathogen [10,19,20]. In wounds that are chronic, especially in patients who have recently been treated with antimicrobial therapy, infections are more frequently polymicrobial and the causative pathogens are more diverse, often including aerobic gram-negative bacilli and obligate anaerobic bacteria [10,21]. The presence of a mixture of bacterial types appears to predispose to the production of virulence factors, such as hemolysins, proteases and collagenases, as well as short-chain fatty acids; these cause inflammation, impede wound healing and contribute to the chronicity of the infection [19,22]. In chronic, clinically uninfected wounds, the presence of some microbes is potentially advantageous, inducing passive resistance, metabolic cooperation, quorum sensing systems and DNA sharing [23].

New data derived using molecular techniques demonstrate that chronic wounds contain many different microorganisms some of which were not previously recognized using standard culture methods. We are only in the early stages of understanding the specific roles these microorganisms play in chronic wounds [23]. Moreover, recent studies from less developed countries, especially in hot, humid climates, report that even with standard microbiological methods aerobic gram-negative bacilli, especially *Pseudomonas aeruginosa* more often cause DFIs [24]. While not yet adequately investigated, these findings are probably related to various environmental, hygienic and cultural issues. To better interpret culture results and provide optimal antimicrobial therapy, clinicians must be familiar with the microbial isolates in their own region of practice. An additional pathogenic property of many organisms is their ability to become enveloped biofilm. This has been best studied in *S. aureus* skin biofilms, which appear to inhibit wound healing, diminish localized immunity and enable other microorganisms to colonize and infect the wound [23]. Furthermore, consortia of genotypically distinct bacteria may symbiotically produce a pathogenic community, referred to as functionally equivalent pathogroups [25].

In the past few decades a major problem in treating DFIs has been the increased rate of isolation of antibiotic-resistant pathogens, particularly methicillin-resistant *S. aureus* (MRSA), and to a lesser degree glycopeptide-intermediate *S. aureus* (GISA), vancomycin-resistant enterococci (VRE), extended-spectrum β-lactamase- (ESBL) or carbapenamase–producing gram-negative bacilli and highly resistant strains of *P. aeruginosa*. The rates of isolation of these multi-drug resistant pathogens vary widely by geographical area and treatment center. But, the potential presence of such resistant isolates emphasizes the importance of obtaining optimal specimens for culture and sensitivity testing for infected DFIs [10,26], as well as avoiding the excessive antibiotic therapy that drives this resistance.

### Evaluation of *S. aureus* virulence genes in DFU/DFI

Staphylococci, in addition to being the most frequent, are perhaps the most virulent pathogens in DFI [10,19,20]. Studies in France have demonstrated a correlation between specific virulence genotypic markers in *S. aureus* isolates from DFU and ulcer outcome [27-29]. Using a miniaturized oligonucleotide array to identify genes encoding resistance determinants, toxins and species-specific sequences of *S. aureus*, Sotto *et al*. sought to differentiate colonized from infected wounds in diabetic patients with a foot ulcer that was culture-positive for only *S. aureus.* Virulence genes were absent in 20 of 22 (92%) clinically uninfected ulcers, but present in 49 of 50 (98%) infected ulcers [27]. In a follow-up study with similar inclusion criteria, these investigators used polymerase chain reaction (PCR) assays to detect genetic markers in both clinically uninfected and infected diabetic foot ulcers. Analyzing for the presence of 31 of the most prevalent virulence-associated *S. aureus* genes they noted that a five-gene combination of capsular type 8 (*cap8*), *Staphylococcus* enterotoxin A (*sea*)*, Staphylococcus* enterotoxin I (*sei*)*,* LukDE leukocidin (*lukD*/*lukE*) and ɤ-hemolysin V (*hlgv*) was most predictive of clinical infection [28]. Then, using a new generation of miniaturized oligonucleotide arrays for genotyping *S. aureus* that covered a larger number of genes, they compared the presence of each gene in *S. aureus* strains to the grades and outcomes of diabetic foot ulcers. Using logistic analyses they found that *lukDE* was the gene most predictive of a favorable outcome of infection resolution or healing of uninfected DFU [29]. These data demonstrate the potential of molecular methods for identifying virulence factors in isolates from DFIs.

### Defining DFI microbiota – a methodological metamorphosis?

Over the past forty years studies to identify pathogens in DFI have used standard microbiological methods, despite their significant time to perform (two to three days for preliminary results with final sensitivities often taking longer), bias in species detected, lack of sensitivity (for example, for fastidious organisms) and lack of information on the relative prevalence of various pathogens and their potential virulence. The recent availability of molecular techniques has shed new light on the microbial world of diabetic foot wounds. They have generally revealed the presence of many more organisms and considerably more species (especially obligate anaerobes) than found with standard cultures. However, molecular methods also have some limitations, including high cost and the need for substantial technician time for some methods. Although advances in technology have produced new desktop sequencers that are easy and somewhat quicker to operate, these machines lack the sequencing throughput required for microbial community sequencing provided by larger-scale sequencers (for example, Life Technology Ion Proton System or Illumina Hiseq X Ten), which are generally available only in large-scale centers. Moreover, the clinical significance of these microbiological findings is as yet unclear [30]. For example, although we advocate selecting as focused an antimicrobial regime as possible, we do not know if antibiotic treatment must be directed at each isolated organism, or only at presumed bacterial ‘ringleaders’, or even at organisms that were once considered probable non-pathogenic ‘lab weeds’ [31]. Thus, to better understand the microbial diversity of wounds in DFI and to identify the relative proportion and types of species in the wound some studies have examined the results of using ‘crime scene investigation (CSI)’-era technology (for example, small subunit ribosomal RNA sequencing methods and real time PCR) to standard culture methods [25,32-34].

#### Standard sample collection and bacterial culture

When obtaining a specimen for culture and sensitivity testing, it is key to collect material that is not contaminated with colonizing flora, but contains the true pathogens. Since prior antibiotic therapy can cause false-negative cultures, it is best if specimens can be obtained before such therapy is begun. In some chronic infections, such as osteomyelitis, it is possible to safely discontinue antibiotic therapy for at least a few days (or even weeks) before obtaining deep cultures [35]. Specimens should be obtained only after cleansing (with non-antimicrobial substances) and debriding the wound. While swabs of open wounds are easy to obtain, most studies comparing them to tissue specimens have shown that they are more apt to grown contaminants and less likely to yield true pathogens [21], especially when sampling bone [36]. Optimal specimens for culture include tissue obtained by curettage of debrided ulcer or a biopsy [37]. It is also important to ensure the specimen is placed in an appropriate sterile transport container, is rapidly sent to the microbiology laboratory and once there is quickly processed.

Despite optimal specimen collection and processing, culture-based techniques select for species that flourish under typical nutritional and physiological conditions of the microbiology laboratory but are potentially not the most abundant or clinically important pathogens [30]. These standard methods may fail to identify slow-growing, fastidious or anaerobic organisms [34]. Performing the 130-year-old method of the Gram-stained smear of a wound specimen can provide rapid information about the presence and type of microorganism and their relative abundance in the tissue. Finally, we now have newer, rapid tests that may provide a more accurate snapshot of the wound microbiological milieu and are widely used in clinical microbiology [7]. But, has the promise of molecular microbiology been fulfilled yet? Let us review concepts of molecular microbiology as a step forward in identifying and better defining the microbiome of DFI.

#### Molecular microbiology

##### PCR

This is a molecular method to amplify a genomic region of interest. When followed by DNA sequencing, the abundance and genetic composition of a gene of interest can be determined. The small subunit (SSU) ribosomal RNA (rRNA) gene in bacteria, called 16S rRNA, is a useful gene target given that it is conserved across all prokaryotes (bacteria) but not eukaryotes (for example, humans). In a clinical specimen, using universal primers for bacteria in highly conserved regions of this gene permits broad-range amplification by PCR of bacterial SSU rRNA genes, but not human host genes. Simultaneously, identifying species-specific hypervariable regions in the 16S rRNA gene allows for taxonomic classification of bacteria [38]. Following amplification by PCR, 16S rRNA gene fragments are sequenced and analyzed using various methods to assess the taxonomic composition and abundance of bacterial communities [25,30,39,40]. Thus, the combination of conserved primer-binding sites and intervening variable sequences facilitates the identification and quantification of microorganisms at the level of genus and species, to permit a better understanding of a DFU microbiome [30].

Currently, 16S rRNA quantitative PCR (qPCR) is used to determine the biodiversity of wounds and estimate bacterial load. Techniques for determining biodiversity include full ribosomal amplification, cloning and Sanger sequencing (FRACS), partial ribosomal amplification with a gel band identification and Sanger sequencing (PRADS), and density gradient gel electrophoresis (DGGE) [40]. Similarly, PCR assay or oligonucleotide array sequence analyses (hybridization of a nucleic acid sample to a large set of probes for gene mapping) can assess virulence-associated genes [28,29,34,41]. Overall, PCR amplification and sequencing allows for the quantification and analysis of specific genes (or genomic regions) of interest.

##### Metagenomics

In the past decade numerous molecular methods have been introduced for detecting microorganisms from clinical specimens in a wound. Perhaps the most revolutionary are those used to sequence DNA directly from a sample, known as metagenomics [42]. Metagenomic methods can potentially provide not only the names of the pathogens present in an infected wound, but information on their virulence and their antibiotic susceptibility patterns (to selected agents in some cases, but to all drugs when needed), all within a time frame that would allow replacing most empirical antibiotic selections with evidence-based therapy [26,41]. This targeted diagnosis is fundamental for preventing the overuse of broad-spectrum antibiotics that is one of the causes of the emergence of bacterial resistance.

Metagenomic techniques allow for the complete characterization of all bacteria, archaea, fungi and viruses within a sample (that is, the microbiome). Studies with this method suggest that cultivable bacteria comprise only a small fraction (<1%) of the total bacterial diversity [43]. These culture independent methods are revolutionizing clinical microbiology by providing a first glimpse into microbial community structure and function relative to human health and disease [44-46]. The introduction of next-generation sequencing (NGS) technologies (for example, 454/Roche pyrosequencing, Illumina and Ion Torrent) [47-49] allows for the generation of DNA sequence data more quickly and at decreased cost, which should lead metagenomics from the ‘research’ realm into the clinical microbiology laboratory. Metagenomic methods differ from other molecular methods in the steps involved to prepare samples for sequencing, time it takes to obtain results, number of sequences generated and bacterial diversity observable [23,40].

**Figure 1:**
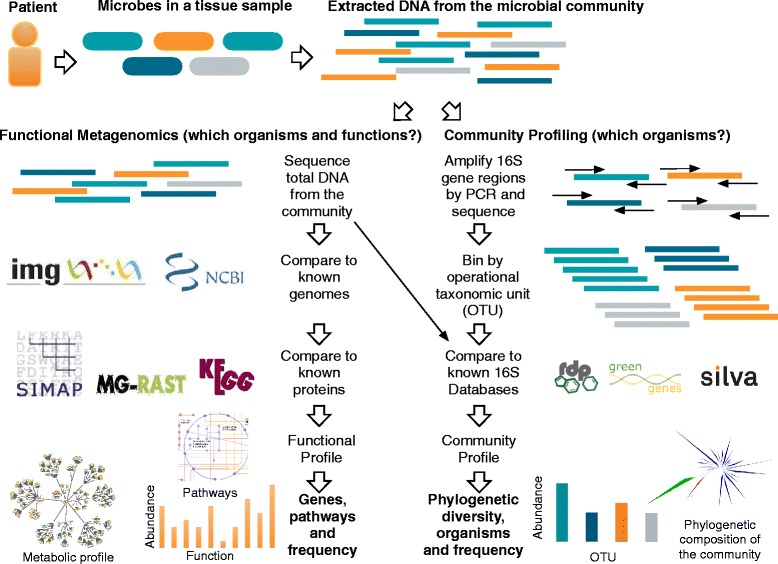
**Overview of methods for community profiling and functional metagenomics.** Patient tissue samples contain a mixture of human and microbial DNA. Microbial DNA is derived from a community of bacteria and other organisms present at their relative abundance in the sample, indicated here using different colors. Once DNA has been extracted from the sample, two metagenomic methods can be applied. In ***functional metagenomics*** the total DNA is sequenced and analyzed by comparing it to databases of known genomes (for example, NCBI and IMG) and 16S rRNA genes (for example, RDP, Green Genes and Silva) to identify bacterial taxa and their abundance. Sequences are also compared to known proteins (for example, SIMAP, MG-RAST, KEGG) for functional analysis of genes, pathways and relative frequency. In ***community profiling***, hypervariable regions of the 16S rRNA gene from bacteria are amplified and sequenced. Highly similar sequences are binned by operational taxonomic units and compared to databases of 16S rRNA genes from known bacteria (for example, RDP, Green Genes and Silva) to identify bacterial taxa and their frequency. 16S rRNA gene sequences can be used in subsequent analyses of phylogenetic diversity in the sample. IMG: Integrated Microbial Genomes; KEGG: Kyoto Encyclopedia of Genes and Genomes; MG-RAST: Metagenomic Rapid Annotations using Subsystems Technology; NCBI: National Center for Biotechnology Information; OTU: Operational Taxonomic Unit; RDP: Ribosomal Database Project; SIMAP: Similarity Matrix of Proteins.

There are now two metagenomic methods for analyzing the microbes within a specimen, that is, community profiling (using a single gene assay such as 16S rRNA) and functional metagenomics (using total DNA), as shown in Figure 1 and further described in Table 1 [50]. Metagenomic community profiles are produced by amplifying regions in the SSU rRNA gene from genomic DNA in a clinical sample and can be more accurate than culture-based approaches [51,52]. Conversely, emerging functional metagenomics methods provide a comprehensive look at bacterial communities by sequencing all genomic DNA in a sample rather than a single gene, such as the 16S rRNA gene [53]. This allows for the characterization of bacteria and their biological processes, including pathogenicity islands (that is, the genetic element of an organism responsible for its capacity to cause disease), virulence factors, and antibiotic resistance [54]. Furthermore, organisms can be classified with better taxonomic resolution than single gene assays, such as 16S rRNA [44,55].

**Table 1:** **Metagenomic methods: community profiling versus functional metagenomics**

**Common Features**	**Unique to community profiling**	**Unique to functional metagenomics**
DNA obtained directly from a wound sample; culture independent	Marker genes such as 16S rRNA gene from a community of bacteria, or 18S rRNA and internal transcribed spacer (ITS) genes for fungi	Can provide genomic DNA from a community of microorganisms. Applicable to all microorganisms including bacteria, fungi, viruses and archaea
Need to remove human host DNA contamination	PCR primers amplify only marker gene fragments from targeted microbes, excluding DNA from the human host	To avoid biases human DNA must be removed after sequencing through computational methods
Sequenced using high-throughput sequencing technologies	Sequencing errors in highly conserved marker genes can lead to incorrect species assignment	Taxonomic assignment based on multiple genes from genomic DNA can lead to more accurate taxonomic community profiles
Reduction in the overall cost of sequencing	Sequencing is directed at only microbial marker genes, making sequencing more cost effective	Sequencing can be more cost-prohibitive due to human host contamination (approximately 90% of DNA in wound samples)
Data represent a community of microorganisms and reflect organismal diversity and abundance	Community profile is based only on taxonomy	Community profile is based on taxonomy and function, indicating the metabolic potential of a microbial community
Less than 1% of organisms are known, leading to incomplete annotation	Closely-related organisms are indistinguishable based on marker gene sequences alone. Not all bacteria are represented in databases of known 16S rRNA genes or 18S and internal transcribed spacer (ITS) for fungi	Not all microbial genomes exist in databases of known species leading to difficulty in assigning sequences to discrete organisms
Has potential for serendipitous discovery of clinically relevant organisms or function	Novel variations in the hypervariable regions of marker genes can indicate new species	Genomes of unknown organisms can be reconstructed from genomic fragments in metagenomes, providing insights into new species and function
Has potential to find human-microbe interactions	Can find links between microbial community composition and clinical factors or patient outcomes	Can find links between microbial community composition and function and clinical factors or patient outcomes

#### Limitations of molecular microbiology

Despite their great possible clinical utility, each of these molecular techniques (especially PCR based and functional metagenomics) has recognized pitfalls that may block translation from research laboratory to clinical practice. Overall, diagnostic tests based on PCR are subject to issues related to detection sensitivity and specificity, which may lead to an inaccurate portrayal of bacterial communities in wounds [56-58]. Specifically, PCR amplification of genomic fragments requires that PCR primers are unique, bind specifically to a region of interest and bind efficiently enough to produce a PCR product. Given these criteria, PCR primer design relies on *a priori* knowledge of genomic sequences of bacteria in a wound (that may not be cultivable and, therefore, amenable to genome sequencing) and may not be broad enough to account for natural variation in bacteria in polymicrobial wound samples. As a result, genomic fragments that are amplified by PCR may be affected by primer bias, leading to inaccurate representation of the bacterial community.

Even when PCR is successful, the targeted gene must have enough discriminatory power to differentiate related microorganisms. In particular, community profiling based on single gene assays, such as 16S rRNA, may yield inconclusive results for closely related species that lack variation in this highly conserved gene. Moreover, diagnostics based on a single gene, such as 16S rRNA, are limited to bacterial community composition analysis, thereby failing to capture clinically important functional information, such as on virulence factors or antibiotic resistance genes. Lastly, errors in sequencing, such as well-documented issues with homopolymer regions in 454/Roche pyrosequencing [59], could lead to misrepresentation of bacterial communities.

In contrast to 16S rRNA community profiling, functional metagenomics holds great promise in assessing DFI, given that: sequencing is unbiased, allowing for accurate measurement of species and bioburden in the sample; it can be used for viruses that lack conserved genes, such as 16S rRNA; it can be used to analyze multiple genes at one time, including virulence and antibiotic resistance factors; and, it can be used to discover new pathogens or virulence factors. Major concerns with this approach are that datasets are more costly to produce and larger and more complex to analyze. Moreover, it is challenging to separate human-host DNA from microbial DNA in skin samples and to obtain enriched microbial genomic DNA for sequencing [60]. Of the total DNA in a skin sample, approximately 90% is human. Thus, defining microbial DNA requires deep sequencing at a higher cost, with subsequent *in silico* (computer) removal of human sequence contaminants, making functional metagenomics not conducive to rapid diagnosis. Because of this limitation all studies to date on DFU and DFI have been based entirely on 16S rRNA community profiling.

**Figure 2:**
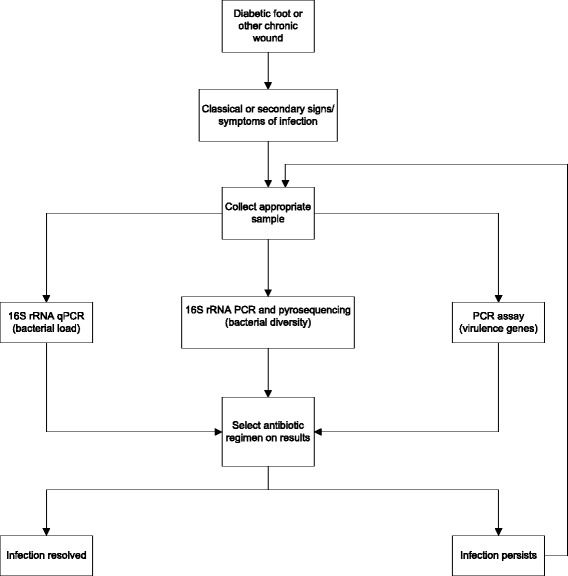
**Proposed algorithm for diabetic foot or other chronic wound infection management using molecular microbiology methodology.**

In Table 2 we have summarized the potential advantages, selected disadvantages and costs of molecular methods that are more apt to be taken up in the clinical microbiology laboratory (metagenomic community profiling, qPCR and virulence genes assays). Given limitations associated with the various methods described, we propose a three-pronged molecular approach: 1) identification of bacterial diversity; 2) quantification of microbial load; and 3) identification of virulence factors of any *S. aureus* isolates. This methodology may allow for a rapid and broader understanding of the microbiological factors affecting a DFU (see Figure 2).

**Table 2:** **Key features of molecular methods for characterizing microorganisms from a diabetic foot infection**

**Molecular Method**	**Potential Advantages**	**Potential Disadvantages**	**Time to results**	**Current cost**
Bacterial identification and quantification^a^				
PCR and pyrosequencing	Delineates full array bacteria present, including almost all gram-positive, gram-negative and obligate anaerobic species; allows broad-range amplification by PCR; detects even small concentrations of microorganisms; avoids false-negative results related to recent antibiotic therapy; can help differentiate colonization from infection	Identifies only 16S bacteria; fails to detect some bacterial and nonbacterial microorganisms; cannot reliably distinguish between viable and nonviable organisms as it amplifies dormant or dead bacteria; unable to test for phenotypic antibiotic sensitivity	4 to 24 hours	About US $13/ target region
q PCR assay^a^	Measures the quantity of a target sequence; determines the number of DNA copies in a sample; estimates bacterial load; helps differentiate colonization from infection	Quantifies DNA from both viable and nonviable bacteria; requires a well-equipped laboratory with PCR facilities	2 to 6 hours	About US $10 per sample
Virulence genes factors for *S. aureus* ^b^				
PCR assay	Allows virulence genotyping among strains of *S. aureus*	Only patients with monomicrobial culture for *S. aureus* were included in published study	2 to 5 hours	About US $5/assay
DNA microarray	Carries a set of 334 different probes for genotyping *S. aureus* isolates; analyzes a large number of samples (96/well strip)	Only patients with monomicrobial culture for *S. aureus* were included in published study	4 to ~5 hours	About US $ 60/96-well strip

^a^Indicates that these molecular methods used 16S rRNA gene. In this table we decided to present one of methods (the newest) that have been used for identification of bacterial diversity in the diabetic foot infection: PCR and pyrosequencing instead of other methods, such as PCR and Sanger sequencing. ^b^Indicates that these methods have been used for differentiating colonization from infection and non infected from infected ulcer in diabetic foot ulcer.

**Figure 3:**
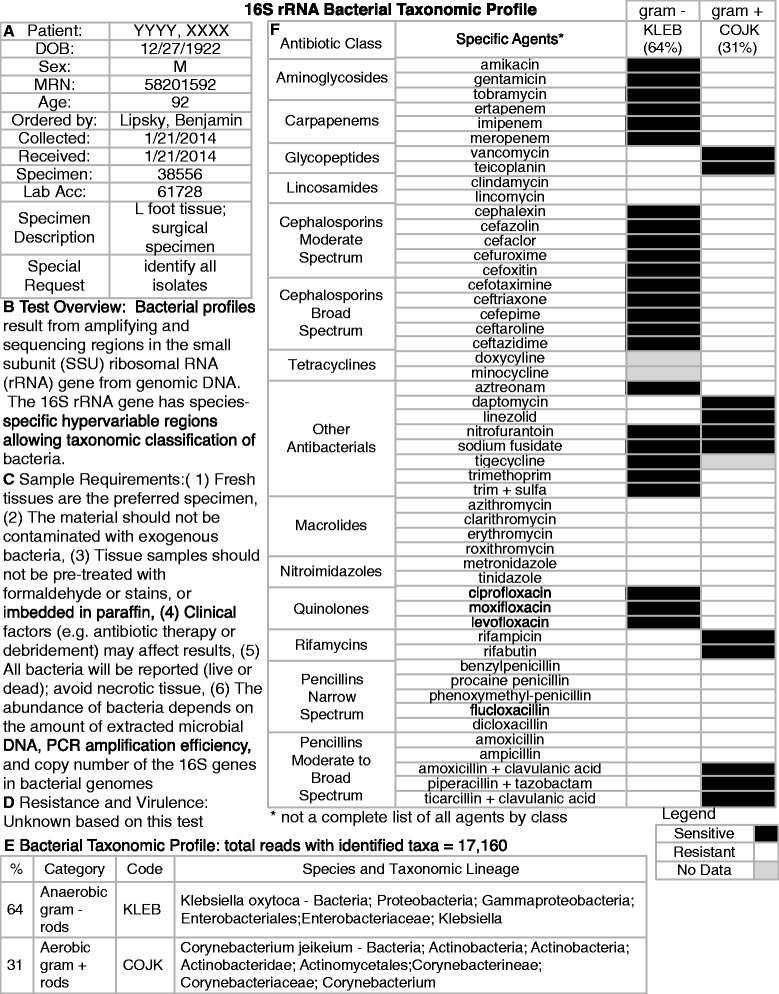
**Example of a potential microbiology report produced using the results of 16S rRNA (NGS) data.** Example of a potential microbiology report produced using the results of 16S rRNA NGS data from an actual patient specimen from the Southern Arizona Limb Salvage Alliance clinic. **A)** Patient and specimen information, **B)** Test description and overview, **C)** Sample preparation requirements, **D)** List of any resistance or virulence factors detected (note that this test does not yield these data), **E)** Bacterial taxonomic profile, **F)** Antibiotic susceptibility profile based on the bacterial taxa detected in this sample. NGS, next-generation sequencing.

**Figure 4:**
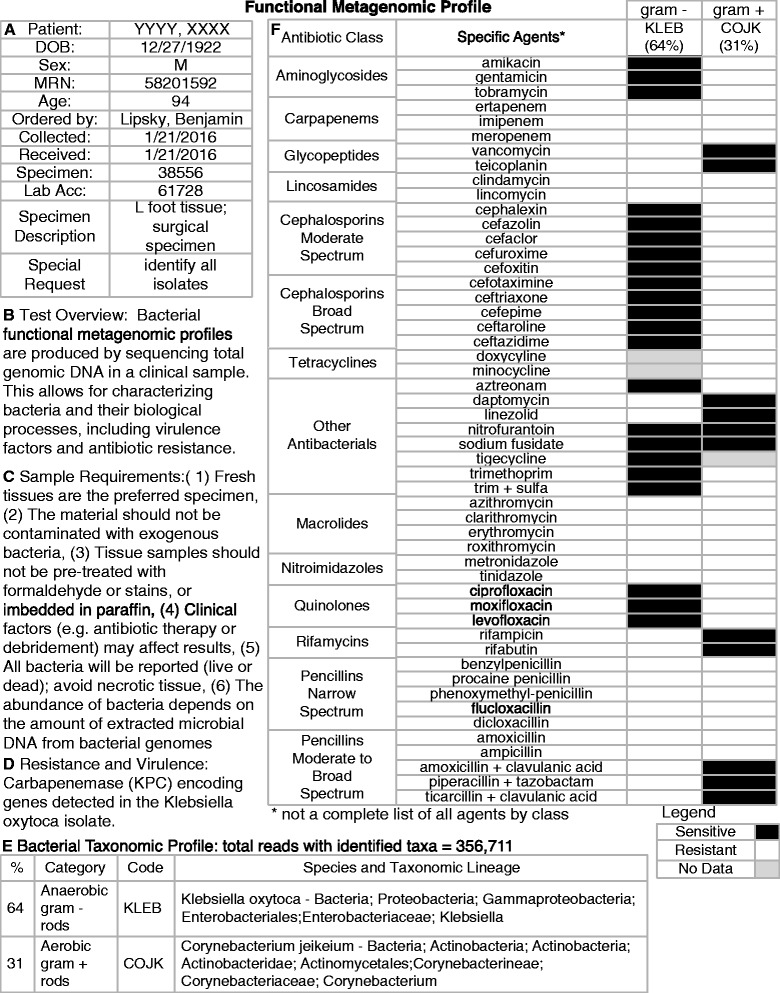
**Example of a potential microbiology report based on hypothetical functional metagenomic next generation sequencing (NGS) data.** Example of a potential microbiology report based on hypothetical functional metagenomic NGS data from a patient specimen. **A)** Patient and specimen information, **B)** Test description and overview, **C)** Sample preparation requirements, **D)** List of any resistance or virulence factors detected, **E)** Bacterial taxonomic profile, **F)** Antibiotic susceptibility profile based on bacterial taxa detected and antibiotic resistance and virulence factors detected.

Finally, translating large-scale metagenomic datasets into a clinical report requires the synthesis of bacterial abundance, virulence factors and antibiotic resistance towards understanding the full susceptibility profile of pathogens. As such, we speculate on what a report might look like based on community profiling (Figure 3) and functional metagenomics (Figure 4) to demonstrate the increased resolution that clinicians might expect to see when moving from Pasteur to CSI.

#### Microbial diversity and bacterial load in patients with DFU: molecular versus culture techniques

Several recent studies have compared standard culture to molecular community profiling techniques to assess the effectiveness of each approach in characterizing bacterial diversity and microbial load in DFU and DFI [34,40,61]. Although the types of wounds and methodology differed in the studies, we will focus our discussion on the analysis of diabetic foot ulcers.

Dowd *et al*. performed a comprehensive survey of bacterial diversity on three groups of patients with chronic wounds with pathogenic biofilms, including one group with DFU [40]. Analyses of a single pooled sample of the 10 patients in the DFU group for 16S partial ribosomal amplification and 454/Roche pyrosequencing generated approximately 36,000 sequences. Rhoads *et al*. compared results of parallel samples processed by aerobic culture versus 16S rRNA partial ribosomal amplification and 454/Roche pyrosequencing from 168 patients with chronic wounds, including 40 on the lower extremity of diabetic patients [61]. Gardner *et al*. compared the results detected by community profiling versus culture of three dimensions of DFU bioburden (microbial diversity, microbial load and pathogenicity) in 52 patients with DFUs. Microbial diversity was defined as the number of bacterial taxa present using 16S rRNA community profiling and microbial load was defined as the total quantity of microbes present using quantitative real time PCR. Sequences were assigned to operational taxonomic units (OTU), molecular proxies for describing organisms based on their phylogenetic relationship to other organisms. Because pathophysiologically distinct DFUs likely lead to confounding identification of microbial diversity, all 52 subjects selected had only a specific homogeneous type of wound, that is, a neuropathic nonischemic DFU. Roche/454 pyrosequencing showed an average of 5,634 sequences generated per sample [34].

These studies reported somewhat different taxonomic compositions of DFUs. Dowd *et al*. found the primary bacterial genera were *Staphylococcus* (29.7%)*, Peptoniphilus* (6.9%)*, Rhodopseudomonas* (6.9%) and *Enterococcus* (6.4%) [40]. Facultative and strictly anaerobic gram-positive cocci were the most prevalent isolates. They used two traditional methods: FRACS showed the overwhelmingly predominant species was *S. aureus*, followed by *Anaerococcus lactolyticus, Anaerococcus vaginalis, Bacterioides fragilis, Finegoldia magna and Morganella morganii*; PRADS identified *Pseudomonas, Haemophilus, Citrobacter* and *Stenotrophomonas* as the predominant species. The molecular methods differ in the number of sequences generated and the variety of bacteria found with different physiological and phenotypic preferences. Molecular methods identified all bacterial isolates found on standard culture, but they were performed during the study, while the cultures were reviewed retrospectively [40].

In the Rhoads *et al*. study, the most common genera detected in DFU by molecular testing were *Corynebacterium*, *Peptoniphilus*, *Staphylococcus*, *Anaerococcus* and *Bacteroides*, while the most frequent on culture were species of *Enterococcus*, *Staphylococcus*, *Pseudomonas*, *Serratia* and *Proteus* [61]. Finally, Gardner *et al*. detected a total of 13 phyla, with the majority of sequences being Firmicutes (67%), Actinobacteria (14%), Proteobacteria (9.8%), Bacteroidetes (7.3%) and Fusobacteria (1.4%). The most abundant OTU, *Staphylococcus*, comprised 29% of all sequences. Culture results showed a much higher relative abundance of *Staphylococcus* (46%) and a much lower prevalence of anaerobic bacteria (12%). Furthermore, cultures substantially underestimated the bacterial load based on qPCR of the 16S rRNA gene by an average 2.34 logs and, in some cases, by more than 6 logs [34].

Taken together, these three studies strongly suggest that molecular techniques, such as 16S rRNA community profiling, identify a greater diversity of organisms than do standard microbiological methods. In particular, they reveal more fastidious anaerobes and gram-negative species than previously recognized. These results have been affirmed in clinical case studies that have demonstrated the potential utility of 16S rRNA community profiling over culture [62].

### Translating new technologies for bacterial identification to the clinic - a possible future use in DFI?

New point-of-care (POC) testing methods may be useful in a variety of clinical settings. The ideal diagnostic test would be accurate, portable, low cost, and require minimal technical skills. POC testing could be used by the patient (or care-giver or visiting nurse) at home in selected circumstances, and could also help determine the level of care (in the ambulatory or inpatient setting) needed by a patient. In evaluating a diabetic foot wound in the outpatient setting, POC testing could provide a mechanism for early detection of infection, allowing clinicians to determine which wounds to culture and to provide definitive (rather than empiric) antibiotic therapy before the patient leaves the clinic [63]. We can (or soon will be able to) get all of this clinically useful information in ‘real’ time, before the clinician sits down to write orders for further microbiological testing or antibiotic treatment. The United States Food and Drug Administration (FDA) has already approved rapid antigen tests for a variety of selected pathogens. While viral assays currently have the lion’s share of these approvals [63] it is likely they will be increasingly used for bacterial identification, including for diagnosing DFI pathogens. Advanced microbiology diagnostic tests provide the promise of dramatically increased sensitivity of pathogen identification in decreased time.

## Conclusions

We are at a critical juncture in the diagnosis of communicable diseases. With the advent of new molecular technologies we can now detect and monitor many pathogens much more rapidly and accurately than with the clinical microbiology methods largely developed 150 years ago by Pasteur. While culture-based techniques have served us well, overcoming their deficiencies will afford us the ability to determine who needs antimicrobial therapy, as well as to quickly select the most appropriate treatment regimen. The currently available data suggest that the promise of molecular microbiology is on the verge of being fulfilled. But, like almost all technological breakthroughs, from tanks to transistors, we must learn how best to use them. Specifically, we will need to carefully evaluate these new methods to better understand if the extra data they provide is clinically useful. If so, we will need to ensure their proper translation into the clinical microbiology laboratory and clinical settings. The ability to integrate data from 16S rRNA PCR, NGS, qPCR and virulence factor detection from a sample collected from a diabetic foot wound should lead to more accurate diagnosis and targeted antibiotic therapy. We should soon be able to put a swab/probe/new device into a cleaned wound and get a report on which organisms are present, in what amounts, with what virulence and antibiotic resistance genes-- all within an hour (or less). In this way, we will indeed be transitioning from ‘Pasteur to CSI.’

***“Gentlemen: It is the microbes who will have the last word”***

***-Louis Pasteur* [**1**]**

***“We solve these cases regardless of race, color, creed, or bubblegum flavor!”***

***- Grissom, “CSI”* [**64**]**
